# Redefining the applications of navigation in spine surgery

**DOI:** 10.3171/2026.4.FOCVID2631

**Published:** 2026-07-01

**Authors:** Daniel Faraj, Michael Galgano

**Affiliations:** Department of Neurosurgery, University of North Carolina School of Medicine, Chapel Hill, North Carolina

**Keywords:** spine surgery, navigation, operative efficiency, ultrasonography

## Abstract

Intraoperative navigation in spine surgery has evolved into an increasingly versatile platform that enhances precision, safety, and operative workflow across a variety of surgical procedures. In the setting of burst fractures, it improves spatial awareness during vertebrectomy, ensuring complete disc removal, accurate endplate preparation, and safe anterior column reconstruction. In sacral and metastatic tumors, navigated osteotomies facilitate precise margin definition and tumor resection. Navigated deployment of interbody and expandable corpectomy cages optimizes positioning and reduces fluoroscopy dependence. Finally, ultrasonography allows for real-time visualization of tools in trauma cases. These instances collectively underscore the multifaceted role of navigation beyond instrumentation alone.

The video can be found here: https://stream.cadmore.media/r10.3171/2026.4.FOCVID2631

## Transcript

Within the upcoming video, we plan to use several clinical cases to highlight the expanded application of navigation in spinal surgery. One of the first ways that we harness the power of spinal navigation during surgery is through the concept of spatial awareness.

### 0:39 Case 1: Background.

This is a CT scan of an unfortunate gentleman who presented to our facility after a fall down several steps. He was found to have a compression fracture at T10 and a burst fracture at T12 with retropulsion into the spinal canal. Clinically, he presented to us with an incomplete spinal cord injury. Due to the nature of the injury and radiological findings, we decided to perform a posterior T12 vertebrectomy with multiple levels of instrumentation above and below his fracture.

### 1:09 Case 1: Surgical Approach and Use of Navigation.

In this case, we specifically used navigation to assist us in finding the disc space above and below the index level fracture site. Performing a discectomy above and below the fracture level is a critical part of this operation. Until the disc is completely removed, we are unable to be confident that the endplates above and below the fracture level have been completely cleared of any disc.

A very common mistake that is made early in practice is to neglect taking down the inferior and superior endplates of the index fracture level. Once again, the navigation can be harnessed in this situation to verify that we are, in fact, at the superior endplate of L1 and inferior endplate of T11, which are the endplates that must be in contact with our expandable corpectomy cage later on in the operation. Here, we are using a 6-mm coarse diamond drill bit to create a cavity within the T12 vertebral body. This is being done in an effort to create a small space for which we can tamp forward any of the retropulsed fracture fragments, as can be seen here. A Woodson elevator is used to create a space ventral to the dura, and a PSO impactor can then subsequently be passed to the contralateral side and gently tamped in the ventral direction, fracturing any of the retropulsed fragments into our cavity. We then use a pituitary rongeur to remove any of the remaining bony fragments, as well as disc material at the T11–12 and T12–L1 levels.

Prior to cage deployment, it is imperative that the endplates are completely cleaned of disc material and cartilage. Once again, we are using navigation here to verify our depth within the T12 vertebral body. As we continue our vertebrectomy in the anterior direction, it is incredibly important that we do have a confident understanding as to how far away we are from the vascular structures that reside anterior to the vertebral column. Although fluoroscopy can also be used for this purpose,^1^ we find that navigation tends to be more efficient as a continuum for verification prior to expandable cage deployment.

### 3:56 Case 2: Sacrectomies and Navigation.

We have also found navigation to be very useful during sacrectomies. In this particular case, the chordoma extends to the surface of the skin. We utilize our navigation to map out the borders of the tumor prior to making the incision. This is a case of a patient with rectal cancer and contiguous extension to the ventral sacrum. Here, we are harnessing the power of using a navigated drill to ensure that we create a very precise ventral osteotomy, just rostral to the extension of the neoplastic tissue into the anterior sacrum. This allows us to maximize our precision prior to tumor delivery.

### 4:44 Case 3: Utilizing Navigation for Interbody and Corpectomy Cages.

The second way that we have expanded the application of navigation in spine surgery is through the deployment of both interbody and corpectomy cages with navigated guidance. In this particular patient with severe spinal stenosis and a grade 1 degenerative spondylolisthesis, we decided to offer the patient a minimal access transforaminal lumbar interbody fusion.^2,3^ Pedicle screws are obviously the traditional indication for spinal navigation. However, in this particular case, after placing our screws with navigated guidance, we decided to use navigated instruments to perform our discectomy as well as for our cage trials. After the cage trials are used to select the appropriately sized expandable cage, we could then subsequently place the cage itself under navigated guidance rather than using fluoroscopy. We then subsequently confirmed our cage placement with x-ray at the end of the operation.

### 5:53 Case 4: Surgical Approach and Considerations.

In this next case, we can appreciate the presurgical MRI and CT scan of a patient with prostate cancer presenting with significant neurological deficits and mechanical-type back pain. He was found to have a pathological fracture at the level of L2 with retropulsion into the spinal canal.

We decided to perform a posterior L2 corpectomy. After we have performed an L2 laminectomy as well as taken down the inferior lamina of L1, we then subsequently perform facetectomies, rostral and caudal to the index level. The L2 nerve roots are then exposed beyond the dorsal root ganglion, and we use angled instruments to tamp down any of the pathologically retropulsed bone fragments. A high-speed drill is then used to resect any of the remaining bone within the cavity of the L2 vertebral body. In our experience, one of the more time-consuming components of the posterior corpectomy is ensuring that the expandable cage is perfectly positioned both in the AP and lateral planes on fluoroscopy. Using x-ray during this part of the operation can also be very ergonomically uncomfortable for the surgeon as well. This generally holds true because the C-arm is a space-occupying structure within the surgical field. We have also found that both the patient and staff are subjected to a rather significant amount of radiation during this part of the operation.^4^

### 7:32 Case 4: Use of Navigation for Cage Insertion.

Alternatively, the surgeon can use a navigated corpectomy cage inserter. As we are turning the corner ventral to the thecal sac, we can appreciate on the navigation screen that the cage itself is being positioned perfectly in the AP and lateral planes prior to cage expansion and final deployment. We have found this to be a very advantageous application of navigation during corpectomy cases.

### 8:07 Ultrasonography: Background.

Ultrasonography has traditionally been regarded as an intraoperative imaging adjunct.^5,6^

We have come to realize over the last several years that ultrasonography can also be used as a means of real-time navigation during spine surgery.

### 8:26 Case 5: Background and Surgical Considerations.

In this particular case, a patient presented to our emergency department with a severe cauda equina injury from an L2 burst fracture with retropulsion into the spinal canal. Despite having performed a laminectomy, intraoperative ultrasound confirms that there is still a large retropulsed bone fragment impeding upon the ventral thecal sac and traversing nerve roots.

We decided to perform a ventral impaction rather than the corpectomy in this particular case.

### 9:00 Case 5: Using Ultrasonography for Navigation.

Here, we have decided to harness ultrasonography to visualize our angled curette in real time. This allows us to use real-time guidance to observe our tools anterior to the thecal sac and find any remaining retropulsed bony fragments that are impeding upon the ventral thecal sac and traversing nerve roots. There are times during such cases where a small bony fragment remains, which can sometimes be very difficult to visualize from a posterior corridor. Harnessing the power of ultrasonography allows us to visualize this remaining bony fragment in real time to ensure that it is properly and safely impacted into the ventral space. Postoperative imaging reveals reconstitution of the L2 vertebral body as well as clearance of the spinal canal.

### 10:08 Conclusion.

In conclusion, we hope this video presentation has given the audience a new fund of knowledge in regard to some of the expanded applications of navigation in spinal surgery.

## Disclosures

The authors report no conflict of interest concerning the materials or methods used in this study or the findings specified in this paper.

## Author Contributions

Primary surgeon: Galgano. Editing and drafting the video and abstract: both authors. Critically revising the work: both authors. Reviewed submitted version of the work: both authors. Approved the final version of the work on behalf of both authors: Faraj. Supervision: Galgano.

## Supplemental Information

### Patient Informed Consent

The necessary patient informed consent was obtained in this study.
